# The agonistic TSPO ligand XBD173 attenuates the glial response thereby protecting inner retinal neurons in a murine model of retinal ischemia

**DOI:** 10.1186/s12974-019-1424-5

**Published:** 2019-02-18

**Authors:** Kristin Mages, Felix Grassmann, Herbert Jägle, Rainer Rupprecht, Bernhard H. F. Weber, Stefanie M. Hauck, Antje Grosche

**Affiliations:** 1 Institute of Human Genetics, University of Regensburg, Franz-Josef-Strauß-Allee 11, 93053 Regensburg, Germany; 2 Department of Medical Epidemiology and Biostatistics, Karolinska Institutet, Nobels väg 12A, Stockholm, Sweden; 3 Department of Ophthalmology, University of Regensburg, Franz-Josef-Strauß-Allee 11, 93053 Regensburg, Germany; 4 Department of Psychiatry and Psychotherapy, University of Regensburg, Universitätsstraße 84, 93053 Regensburg, Germany; 5 Research Unit Protein Science, Helmholtz Zentrum München, German Research Center for Environmental Health (GmbH), Heidemannstraße 1, 80939 Munich, Germany; 6 Department of Physiological Genomics, Ludwig-Maximilians-Universität München, Großhaderner Str. 9, 82152 Planegg-Martinsried, Germany

**Keywords:** Retina, TSPO, Müller cell, Microglia, Gliosis, Ischemia

## Abstract

**Background:**

Ligand-driven modulation of the mitochondrial translocator protein 18 kDa (TSPO) was recently described to dampen the neuroinflammatory response of microglia in a retinal light damage model resulting in protective effects on photoreceptors. We characterized the effects of the TSPO ligand XBD173 in the postischemic retina focusing on changes in the response pattern of the major glial cell types of the retina—microglia and Müller cells.

**Methods:**

Retinal ischemia was induced by increasing the intraocular pressure for 60 min followed by reperfusion of the tissue in mice. On retinal cell types enriched via immunomagnetic separation expression analysis of TSPO, its ligand diazepam-binding inhibitor (DBI) and markers of glial activation were performed at transcript and protein level using RNA sequencing, qRT-PCR, lipid chromatography-mass spectrometry, and immunofluorescent labeling. Data on cell morphology and numbers were assessed in retinal slice and flatmount preparations. The retinal functional integrity was determined by electroretinogram recordings.

**Results:**

We demonstrate that TSPO is expressed by Müller cells, microglia, vascular cells, retinal pigment epithelium (RPE) of the healthy and postischemic retina, but only at low levels in retinal neurons. While an alleviated neurodegeneration upon XBD173 treatment was found in postischemic retinae as compared to vehicle controls, this neuroprotective effect of XBD173 is mediated putatively by its action on retinal glia. After transient ischemia, TSPO as a marker of activation was upregulated to similar levels in microglia as compared to their counterparts in healthy retinae irrespective of the treatment regimen. However, less microglia were found in XBD173-treated postischemic retinae at 3 days post-surgery (dps) which displayed a more ramified morphology than in retinae of vehicle-treated mice indicating a dampened microglia activation. Müller cells, the major retinal macroglia, show upregulation of the typical gliosis marker GFAP. Importantly, glutamine synthetase was more stably expressed in Müller glia of XBD173-treated postischemic retinae and homeostatic functions such as cellular volume regulation typically diminished in gliotic Müller cells remained functional.

**Conclusions:**

In sum, our data imply that beneficial effects of XBD173 treatment on the postischemic survival of inner retinal neurons were primarily mediated by stabilizing neurosupportive functions of glial cells.

## Background

Müller cells maintain the retinal homeostasis fulfilling a number of functions including ion and cell volume regulation, neurotransmitter recycling, and expression of neuroprotective factors [1–3]. They are highly responsive to pathological triggers, a reaction termed Müller cell gliosis. Its relevance under pathological conditions is still under debate. Some authors postulate gliosis being primarily detrimental for the long-term survival and/or regeneration of neuronal structures, while others point out neuroprotective aspects [4, 5]. Recently, we demonstrated that this Müller cell gliosis includes changes in their electrophysiological properties (e.g., downregulation of Kir4.1 potassium channels) and a concomitant loss of their ability of volume regulation resulting in a constrained ion and volume homeostasis of the Müller cells with likely consequences on other retinal structures [2, 6]. We also showed that a modified gliotic activation leading to a stable Kir4.1 channel function in reactive Müller cells is beneficial for the survival of inner retinal neurons in the postischemic retina [6].

Translocator protein 18 kDa (TSPO) is an integral membrane protein of the outer mitochondrial membrane, although a localization at the plasma membrane and in or at the nucleus has also been suggested [7]. In the central nervous system (CNS), its expression is primarily confined to ependymal cells, microglia, and astrocytes [8–10]. TSPO functions in cell physiology are diverse but still controversially discussed [11–13]. There is evidence for a role in neurosteroidogenesis as it binds cholesterol, the input substrate for the latter, and shuttles it to the inner mitochondrial membrane where neurosteroid biosynthesis is initiated [7, 11]. Additionally, anti-inflammatory effects of TSPO activity [14] and a reduced respiratory activity in neuroblastoma cells upon treatment with the TSPO ligand PK 11195 [15] have been implicated. In line with these findings, TSPO deficiency leads to a reduced respiratory activity in microglia [16]. Even though the physiological function of TSPO awaits unequivocal proof of existing concepts, a body of literature also described neuroprotective effects of TSPO agonists in disease models of the central [17–19] and peripheral [20] nervous system. Consequently, pharmacological modulation of TSPO is a promising tool of neuroprotection.

TSPO expression in the retina was primarily assigned to reactive microglia [21, 22]. Moreover, treatment with the TSPO agonists enhanced allopregnanolone production with concomitantly improved retinal cell survival in an ex vivo model of high-pressure glaucoma [23] and better photoreceptor survival as well as a dampened microglial response in a model of retinal light damage [24]. The endogenous ligand, diazepam binding inhibitor (DBI), was reported to be exclusively expressed by Müller cells [22]. Accordingly, it was speculated about a close interplay of Müller cells and microglia coordinating the retinal immune response via TSPO signaling.

Given the preexisting data demonstrating (i) an upregulation of TSPO in microglia [21, 22] and reactive astrocytes in the brain [9], (ii) a protective effect of agonistic TSPO ligands in damaged peripheral and central nervous system, and (iii) findings that it modulates the glial response pattern in the context of retinal disease [24], we were interested in effects of enhanced TSPO signaling on Müller cells.

## Methods

### Materials

All substances were purchased from Sigma-Aldrich (Taufkirchen, Germany) unless stated otherwise. Papain was obtained from Roche (Mannheim, Germany). Chloromethyl-tetramethyl-rosamine (Mitotracker Orange) was purchased from Molecular Probes (Life Technologies, Carlsbad, CA, USA). For immunofluorescence staining, the following primary antibodies were used: mouse anti-glial fibrillary acidic protein (GFAP; 1:200; G-A-5 clone, Sigma), goat anti-calretinin (1:500, Swant, Marly, Switzerland), rabbit-anti-Aif1 (1:500, Wako Chemicals), mouse anti-glutamine synthetase (1:1000, Merck Millipore, Darmstadt, Germany), rabbit anti-TSPO (1:100, Abcam, Cambridge, UK), and rabbit anti-DBI (1:200, Sigma). As secondary antibodies, we used Cy5-conjugated donkey anti-goat, Cy3-conjugated donkey anti-rabbit, Cy2-conjugated donkey anti-mouse, Cy3-conjugated goat anti-rabbit, and Cy2-conjugated goat anti-mouse. All secondary antibodies were obtained from Dianova (Hamburg, Germany) and applied at 1:200 dilution.

### Animals

All experiments were done in accordance with the European Community Council Directive 2010/63/EU and the ARVO Statement for the Use of Animals in Ophthalmic and Vision Research and were approved by the local authorities (55.2 DMS-2532-2-182). Mice were maintained with free access to water and food in an air-conditioned room on a 12-h light-dark cycle. For all experiments, C57Bl/6J mice at the age of 2–4 months were used.

### Retinal ischemia

Transient retinal ischemia was always induced in one eye by the high intraocular pressure (HIOP) method. Anesthesia was induced with ketamine (100 mg/kg body weight, intraperitoneal (ip); Ratiopharm, Ulm, Germany), xylazine (5 mg/kg, ip; Bayer Vital, Leverkusen, Germany), and atropine sulfate (100 mg/kg, ip; Braun, Melsungen, Germany). The anterior chamber of the test eye was cannulated from the pars plana with a 30-gauge infusion needle, connected to a saline bottle. The intraocular pressure was increased to 160 mmHg for 60 min by elevating the bottle. After removing the needle, the animals survived for a defined time span as indicated for the respective experiments and, subsequently, were sacrificed with carbon dioxide. The contralateral eye was not cannulated and served as internal control. Animals of the XBD173 treatment group received two intraperitoneal injections per day (10 mg/kg body weight XBD173 (Tocris, Wiesbaden-Nordenstadt, Germany) dissolved in DMSO (anhydrous, ≥ 99.9% pure, Sigma Aldrich)) starting 1 day before the surgery and at the day of the surgery. Thereafter, they received a daily injection of 10 mg/kg body weight until the animal was sacrificed for analysis. The vehicle group was injected the same volume of DMSO as if the respective amount of XBD173 would have been administered according to the same time schedule as the XBD173 group.

### Histological and immunohistochemical staining

Enucleated eyes were immersion-fixed (4% paraformaldehyde for 2 h), washed with phosphate-buffered saline (PBS), cryoprotected in sucrose, embedded in Tissue-Tek® O.C.T. compound (Sakura Finetek, Staufen, Germany), and cut in 20 μm sections using a cryostat. Retinal sections were permeabilized (0.3% Triton X-100 plus 1.0% DMSO in PBS) and blocked (5% normal goat serum with 0.3% Triton X-100 and 1.0% DMSO in PBS) for 2 h at room temperature. Primary antibodies were incubated overnight at 4 °C. The sections were washed (1% bovine serum albumin in PBS) and incubated with secondary antibodies (2 h at room temperature). Cell nuclei were labeled with DAPI (1:1000; Life Technologies). Retinal whole mounts were labeled using a similar protocol, except that tissue was permeabilized by higher concentrations of Triton X-100 and DMSO (1% Triton X-100 plus 3% DMSO in PBS), and secondary antibodies were also incubated at 4 °C overnight. Control experiments without primary antibodies showed no unspecific labeling except for the goat-anti-mouse secondary antibody which labeled the blood vessels (not shown). Images were taken with custom-made VisiScope CSU-X1 confocal system (Visitron Systems, Puchheim, Germany) equipped with a high-resolution sCMOS camera (PCO AG, Kehlheim, Germany). Cell nuclei and calretinin-positive neuronal cells were counted in three retinal layers in 150-μm-wide areas of the central retina close to the optic nerve head (optical slice thickness, 1.5 μm) using ImageJ [25].

### Magnetic-activated cell sorting (MACS) of retinal cells

**Fig. 1:**
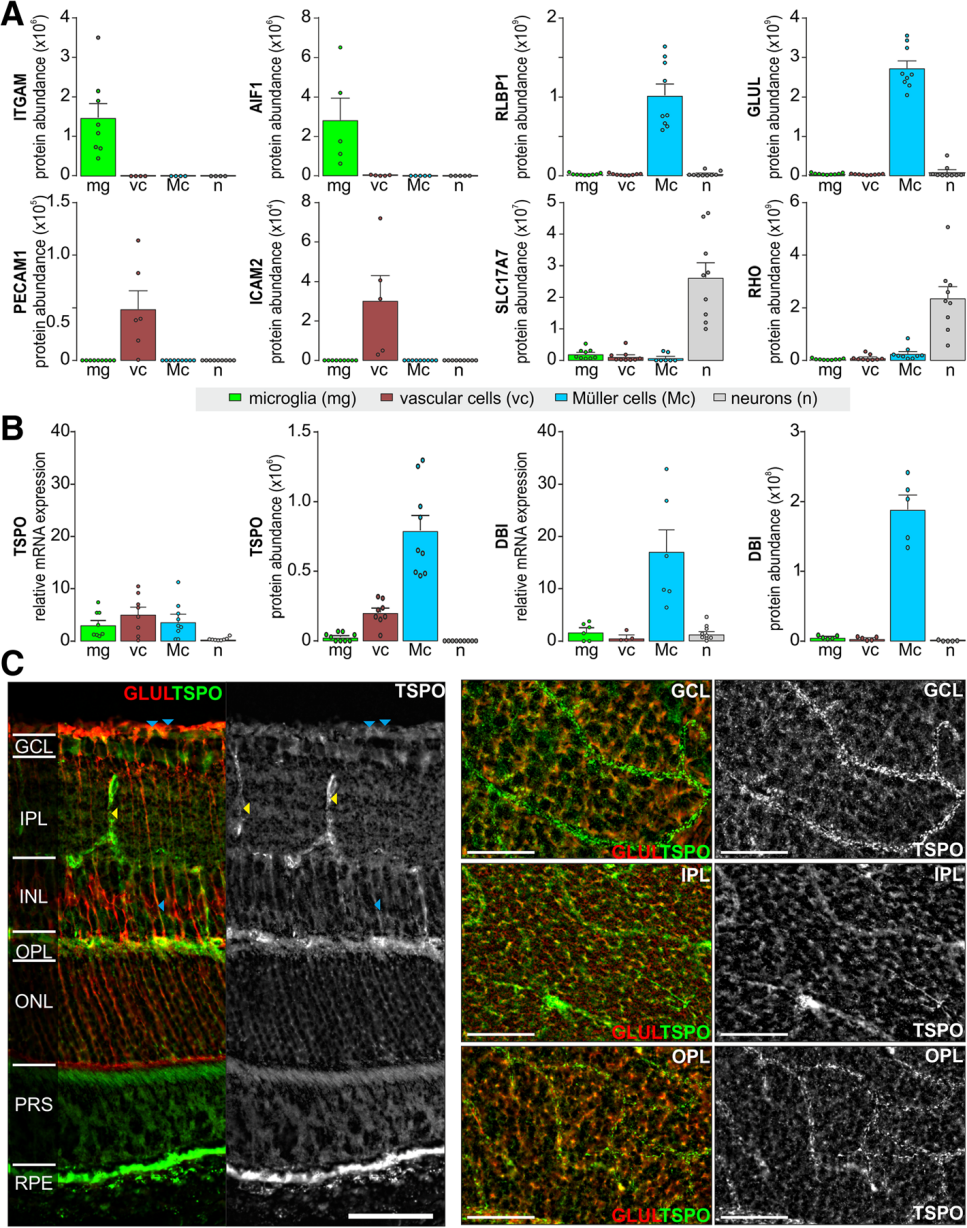
TSPO signaling in different retinal subpopulations. **a** To assess which retinal cell types are directly involved in TSPO-mediated signaling in the healthy murine retina, we enriched microglia, vascular cells, Müller glia, and retinal neurons via immunomagnetic separation. Subsequently, cells were submitted to mass spectrometric analysis to determine protein expression levels. Bar diagrams depict the protein abundance in the respective cell populations for the following cell type-specific marker genes: integrin alpha-M (ITGAM); allograft inflammatory factor 1 (AIF1)—*microglia*; platelet endothelial cell adhesion molecule (PECAM1); intercellular adhesion molecule 2 (ICAM2)—*vascular cells*; retinaldehyde-binding protein 1 (RLBP1); glutamine synthetase (GLUL)—*Müller glia*; vesicular glutamate transporter 1 (SLC17A7); rhodopsin (RHO)—*retinal neurons including photoreceptors*. **b** Transcript and protein expression of TSPO and its endogenous ligand diazepam binding inhibitor (DBI) in the four investigated retinal cell populations. **a**, **b** Values are given as mean ± SEM (*n* = 4–9 biological replicates). **c** TSPO—glutamine synthetase (GLUL) colabeling in retinal slice (left) and flatmount (right) preparations at indicated focus planes. Vessels (yellow arrowheads) and Müller glia (blue arrowheads) are positive for punctuate TSPO labeling indicative of a primarily mitochondrial localization of TSPO. Note the high expression level of TSPO in the retinal pigment epithelium (RPE). GCL, ganglion cell layer; IPL, inner plexiform layer; INL, inner nuclear layer; OPL, outer plexiform layer; ONL, outer nuclear layer. Scale bars, 50 μm

Müller cells were enriched as described previously [26]. Briefly, retinae were digested with papain (0.2 mg/ml; Roche Molecular Biochemicals) for 30 min at 37 °C in the dark in Ca^2+^- and Mg^2+^-free extracellular solution (140 mM NaCl, 3 mM KCl, 10 mM HEPES, 11 mM glucose, pH 7.4). Thereafter, the tissue was incubated with DNase I (200 U/ml) and triturated in extracellular solution containing (mM) 135 NaCl, 3 KCl, 2 CaCl2, 1 MgCl2, 1 Na2HPO4, 10 HEPES, and 11 glucose adjusted to pH 7.4 with Tris. After centrifugation, cells were resuspended and incubated in an extracellular solution containing biotinylated hamster anti-CD29 (clone Ha2/5, 0.1 mg/ml, BD Biosciences, Heidelberg, Germany) for 15 min at 4 °C. Cells were washed in extracellular solution, spun down, resuspended in the presence of anti-biotin MicroBeads (1:5; Miltenyi Biotec, Bergisch Gladbach, Germany), and incubated for 10 min at 4 °C. After washing, CD29+ Müller cells were separated using large cell (LS) columns according to the manufacturer’s instructions (Miltenyi Biotec). To purify microglial and vascular cells in addition to Müller cells, the retinal cell suspension was subsequently incubated with CD11b− and CD31 microbeads (Miltenyi Biotec) and the respective binding cells were depleted from the retinal suspension using LS columns prior to Müller cell enrichment. The cell population depleted from CD11b+, CD31+, and CD29+ cells was considered as the neuronal fraction. However, it needs to be stated that this cell population, we termed “neuronal fraction” in the further manuscript, was negatively selected and thus a minor contamination with other so far unspecified cell types cannot be completely excluded. Given the cellular composition of the retina, the vast majority of these cells however are rod photoreceptors making up about 80% of all retinal cells [27]. As described previously [26], the purity of the CD29+ Müller cell fraction was checked via immunolabeling of the cell suspension for the Müller cell marker glutamine synthetase and a DAPI counterstain to assess total cell numbers. Müller cell purity was above 80% with photoreceptors constituting the main contaminating cell population. Successfully enrichment of the other cell population was validated by expression analysis of marker genes at the protein (mass spectrometry) and transcript (RNA sequencing) level (see Fig. 1).

**Fig. 2:**
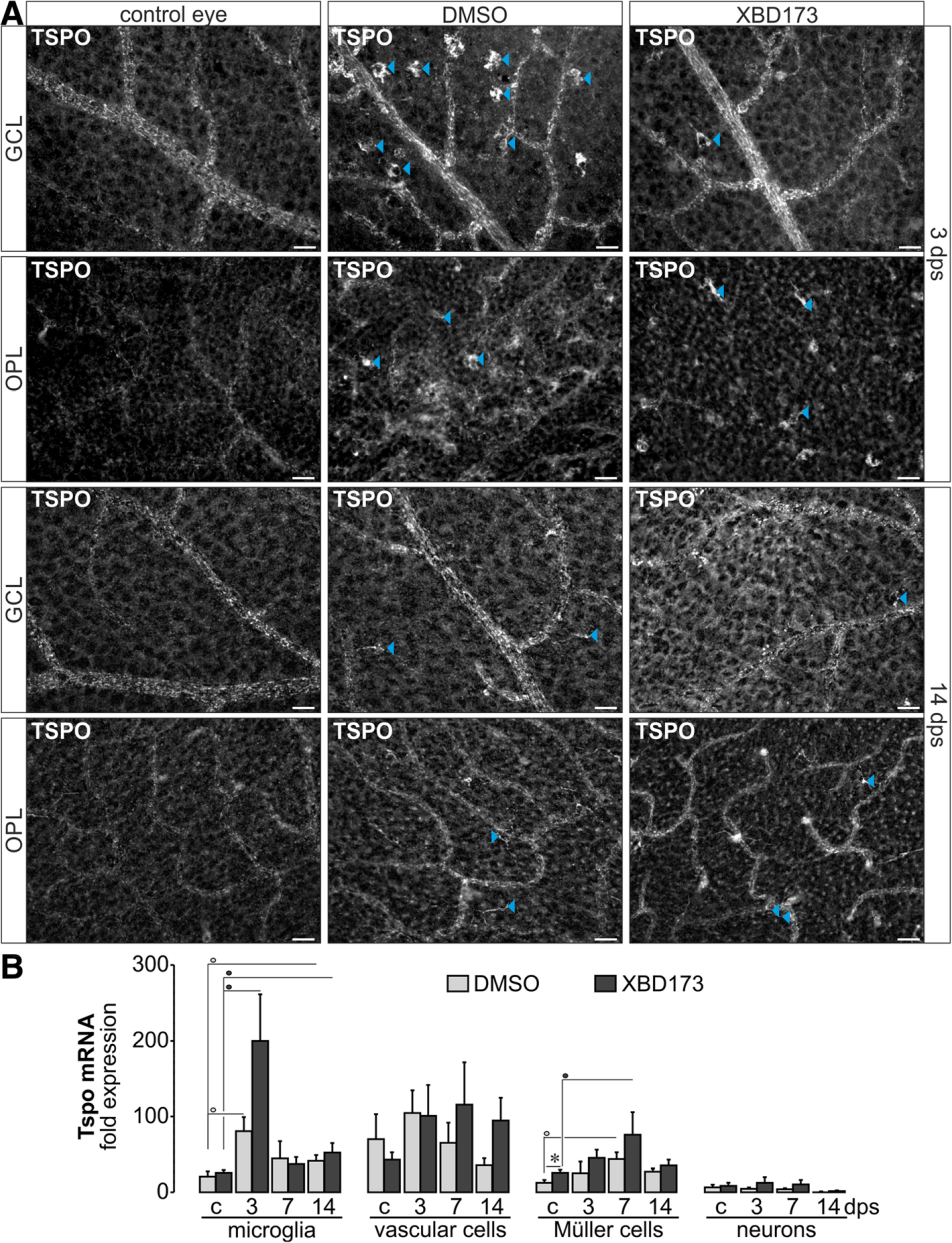
TSPO expression in the postischemic retina. **a** Retinal flatmounts labeled for TSPO focused on the ganglion cell and outer plexiform layer (GCL, OPL) at 3 and 14 dps. While in control retinae primarily endothelial cells and Müller cells appear to be positively labeled, TSPO-positive macrophages/microglia (arrowheads) show up in the postischemic retina irrespective of the treatment regimen. Scale bars, 20 μm. **b** Quantitative real-time PCR (qPCR) performed on retinal cell populations enriched via magnetic activated cell sorting. Bars represent mean values ± SEM (*n* = 3–6 mice of each treatment group and time point). */°/•*P* < 0.05. The color of the circle indicates the treatment group

In sum, none of the cell populations is 100% pure, but needs to be understood as a cell population enriched for a specific cell type like CD31+ vascular cells, CD11b+ microglia and/or invading macrophages (a better discrimination by additional surface markers would lead sample sizes not suitable for the methods used for expression analysis), and CD29+ Müller glia (and few astrocytes). Accordingly, we always investigated the expression of respective genes of interest in all cell populations to get a better overview in which cell types changes in expression levels of genes of interest occur in response to the ischemic insult (see Fig. 2).

### RNA sequencing

After the immunoseparation of retinal cell types, total RNA was isolated from cell pellets using the PureLink® RNA Micro Scale Kit (Thermo Fisher Scientific, Schwerte, Germany). Validation of RNA integrity and quantification was performed using the Agilent RNA 6000 Pico chip analysis according to the manufacturer’s instructions (Agilent Technologies, Waldbronn, Germany). Enrichment of mRNA and library preparation (Nextera XT, Clontech), library quantification (KAPA Library Quantification Kit Illumina, Kapa Biosystems, Inc., Woburn, MA, USA), and sequencing on an Illumina platform (NextSeq 500 High Output Kit v2; 150 cycles) were performed at the service facility KFB Center of Excellence for Fluorescent Bioanalytics (Regensburg, Germany; www.kfb-regensburg.de). After de-multiplexing, a total of at least 20 million reads per sample was reached. Quality control of the reads and quantification of transcript abundance were performed with the Tuxedo suit, as described elsewhere [Pubmed ID26239128 and doi: 10.1007/978-1-4939-8669-9_2]. Briefly, adapter sequences were removed with *cutadapt* [DOI:10.14806/ej.17.1.200], and several quality control measures were queried with *fastqc*. No major problems with the sequencing data were detected. Next, the trimmed reads were aligned to the reference genome/transcriptome (*mm10*) with *HISAT2* [10.1038/nmeth.3317], and transcript abundance was estimated with *stringtie*, expressed as fragments per 1000 bp of transcript per million reads (FPKM).

### qRT-PCR

Like for RNAseq, total RNA was isolated from enriched cell populations using the PureLink® RNA Micro Scale Kit (Thermo Fisher Scientific, Schwerte, Germany). A DNase digestion step was included to remove genomic DNA (Roche). First-strand cDNAs from 10 to 50 ng of total RNA were synthesized using the RevertAid H Minus First-Strand cDNA Synthesis Kit (Fermentas by Thermo Fisher Scientific, Schwerte, Germany). Primers were designed using the Universal ProbeLibrary Assay Design Center (Roche). Transcript levels of candidate genes were measured by qRT-PCR using cDNA with the TaqMan hPSC Scorecard™ Panel (384 well, ViiA7, Life Technologies, Darmstadt, Germany) according to the company’s guidelines.

### LC-MS/MS mass spectrometric analysis

LC-MS/MS analysis was performed as described previously [28, 29] on an Q Exactive HF mass spectrometer (Thermo Fisher Scientific Inc., Waltham, MA, USA) coupled to an Ultimate 3000 RSLC nano-HPLC (Dionex, Sunnyvale, CA). Approximately 0.5 μg per sample were automatically loaded onto a nano-trap column (300 μm inner diameter × 5 mm, packed with Acclaim PepMap100 C18. 5 μm, 100 Å; LC Packings, Sunnyvale, CA) before separation by reversed phase chromatography (HSS-T3 M-class column, 25 cm, Waters) in a 80-min non-linear gradient from 3 to 40% acetonitrile (ACN) in 0.1% formic acid (FA) at a flow rate of 250 nl/min. Eluted peptides were analysed by the Q-Exactive HF mass spectrometer equipped with a nano-flex ionization source. Full-scan MS spectra (from m/z 300 to 1500) and MSMS fragment spectra were acquired in the Orbitrap with a resolution of 60,000 or 15,000, respectively, with maximum injection times of 50 ms each. The up to ten most intense ions were selected for HCD fragmentation depending on signal intensity (TOP10 method). Target peptides already selected for MS/MS were dynamically excluded for 30 s. Label-free analyses (including database search and protein identification was performed as follows. Spectra were analyzed using Progenesis QI software for proteomics (Version 3.0, Non-linear Dynamics, Waters, Newcastle upon Tyne, UK) for label-free quantification as previously described [26]. All features were exported as Mascot generic file (mgf) and used for peptide identification with Mascot (version 2.4) in the UniProtKB/Swiss-Prot taxonomy mouse database (release 2017.02, 16,871 sequences). Search parameters used were 10 ppm peptide mass tolerance and 20 mmu fragment mass tolerance, one missed cleavage allowed, carbamidomethylation was set as fixed modification, methionine oxidation and asparagine or glutamine deamidation were allowed as variable modifications. A Mascot-integrated decoy database search calculated an average false discovery of < 1%.

### Müller cell soma size and volume regulation

Volume changes of Müller cell somata were measured as described previously [30]. Briefly, retinal slices were loaded with the vital dye Mitotracker Orange (10 μM, excitation 543 nm, emission 560-nm-long-pass filter; Life Technologies), which is preferentially taken up by Müller cells [31]. Slices were exposed to a hypotonic solution (60% of control osmolarity using distilled water for dilution) for 4 min. Somata of labeled Müller cells were imaged using confocal microscopy (custom-made VisiScope CSU-X1 confocal system equipped with a high-resolution sCMOS camera; Visitron Systems, Puchheim, Germany), and their cross-sectional areas were measured (ImageJ).

### Full-field electroretinography

Mice were dark adapted for at least 12 h before recordings and anesthetized by subcutaneous injection of ketamine (65 mg/kg) and xylazine (13 mg/kg). The pupils were dilated with tropicamide eyedrops (Mydriaticum Stulln; Pharma Stulln). Silver needle electrodes served as a reference (forehead) and ground (tail) and gold wire ring electrodes as active electrodes. Corneregel (Bausch & Lomb, Berlin, Germany) was applied to keep the eye hydrated and to maintain good electrical contact. ERGs were recorded using a Ganzfeld bowl (Ganzfeld QC450 SCX, Roland Consult, Brandenburg, Germany) and an amplifier with a recording unit (RETI-Port, Roland Consult). ERGs were recorded from both eyes simultaneously, band-pass filtered (1 to 300 Hz), and averaged. Single flash scotopic (dark adapted) responses to a series of ten LED-flash intensities ranging from − 3.5 to 1.0 log cd.s/m^2^ with an interstimulus interval of 2 up to 20 s for the highest intensity were recorded. Responses were quantified based on mean waveform peak amplitude and implicit time. All analysis and plotting were carried out with R 3.2.1 (The R Foundation for Statistical Computing) and ggplot2 2.1.0.

### Statistics

All data are expressed as mean ± standard error (SEM) unless stated otherwise. Statistical analyses were performed using Prism (GraphPad Software, San Diego, CA, USA). In most of the experiments, in the present study, results from four to six biological replicates were collected to keep to the rules of the three Rs for the sake of animal welfare. Since this low number of input values does not allow an appropriate estimation about a normal Gaussian distribution, significance levels were determined by the non-parametric Mann-Whitney *U* test unless stated otherwise.

## Results

### TSPO upregulation in distinct retinal cell types of the ischemic retina

Performing cell type-specific expression analysis at transcript and protein level from microglia, vascular cells, Müller glia, and retinal neurons (Fig. 1a), we found that TSPO is expressed at the highest levels in Müller glia and vascular cells in the healthy neuroretina (Fig. 1b). Immunolabeling for TSPO confirmed these findings and additionally underpinned its robust expression also in the retinal pigment epithelium (RPE) underlying the retina (Fig. 1c). Only little TSPO expression was detected in microglia, particularly if considering protein levels (Fig. 1b).

Next, we investigated the TSPO expression in retinae that had been subjected to transient ischemia (60 min) and subsequent reperfusion. The XBD173 group received intraperitoneal injections starting 1 day before ischemia was induced, while the DMSO group only was injected with the solvent. We found a strong increase of immunoreactivity for TSPO in activated microglia after ischemia as it has been described after light damage [21] (Fig. 2a). There were no obvious changes in the labeling pattern of the other TSPO expressing cell populations (Figs. 2a and 4a). Performing the cell type-specific expression profiling for TSPO mRNA expression in the postischemic retina at different time points after surgery, we found a significant upregulation in microglia of XBD173- and vehicle-treated individuals at 3 days post-surgery (dps) and a subsequent drop of expression to almost baseline levels at 7 dps (Fig. 2b). No significant difference in TSPO regulation in microglia was found between both treatment groups with a tendency of even stronger TSPO upregulation in microglia of XBD173-treated retinae. TSPO transcript expression was slightly but significantly enhanced in Müller glia of XBD173-treated mice already in the healthy control eye and was then significantly upregulated at 7 dps (Fig. 2b), thus few days later as observed in microglia.

### XBD173 modifies the microglial response after transient ischemia

**Fig. 3:**
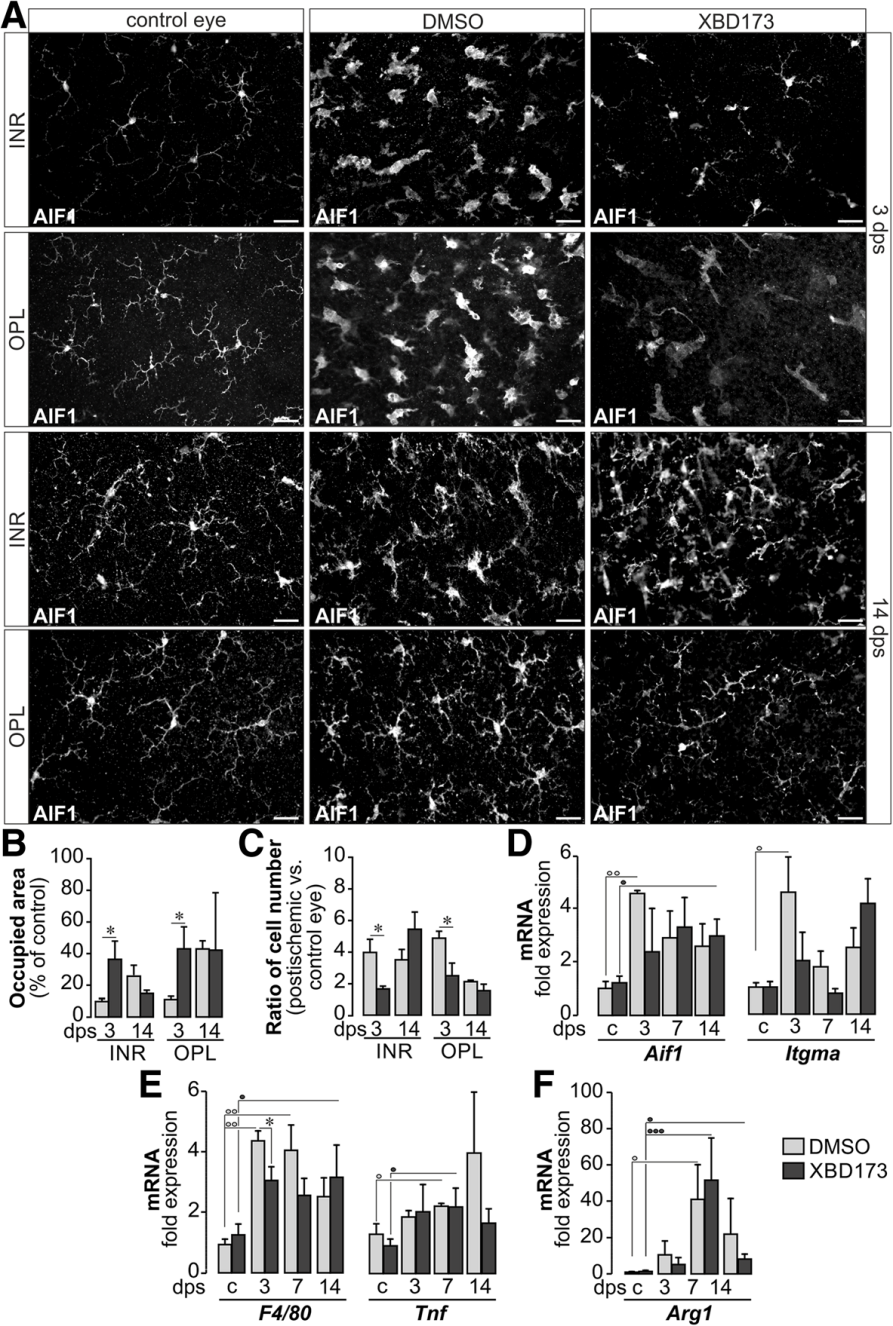
Characterization of the microglial activation pattern in the postischemic retina upon XBD173 treatment. **a** Aif1 labeling of retinal flatmounts delineating microglia in the inner plexiform layer representative of cells quantified in the inner retina (INR) and the outer plexiform layer (OPL) at 3 and 14 dps. Scale bars, 20 μm. **b** Determination of the area occupied by processes of individual microglia as a measure for their activation status. **c** Microglia were quantified (*n* = 4 for each condition) in the OPL and INR (e.g., ganglion cell and inner plexiform layer). **d** Expression levels of Aif1 and Itgam were detected via qPCR. Note that especially 3 days (3) after ischemia, both Aif1 and Itgam expression was the highest in microglia of untreated mice indicative of a stronger microglial activation at this time point Control, **c**. **e** Markers for pro-inflammatory M1 microglia were investigated via qPCR in MACS-sorted cells. TNFα (Tnf) expression was similar in both treatment groups except for microglia 14 days (14) post-surgery. In contrast, less F4/80 was expressed in microglia of XBD173-treated mice. **f** Arginase 1 (*Arg1*) is a marker for anti-inflammatory, protective M2 microglia. Enhanced expression of *Arg1* transcripts may indicate improved neuroprotection in the stressed postischemic retina. **b**–**f** Bars represent mean values ± SEM (*n* = 3–6 mice of each treatment group and time point). */°/•*P* < 0.05; °°*P* < 0.01; •••*P* < 0.001. The color of the circle indicates the treatment group

We went on to investigate other features of microglia activation, since major effects of TSPO agonists on microglia had been described earlier [21, 24]. We found a significantly changed microglial morphology as early as 3 dps. Most cells presented with an amoeboid-shaped phenotype having almost completely retracted their processes (Fig. 3a). This morphological change is reflected by a smaller area occupied by processes of a single microglia. Of note, microglia maintained a slightly more ramified morphology in postischemic retinae of XBD173- compared to DMSO-treated mice (Fig. 3b) at 3 dps. Next, we asked where microglia accumulated in the postischemic tissue and found considerable differences. At 3 dps, twice as many microglia were present in the plexiform layers of postischemic retinae of vehicle controls as compared to the XBD173 group (Fig. 3c). At 14 dps, the differential microglia distribution was no longer noticeable, and the microglial response was declining (e.g., less cells, more ramified morphology) in the outer plexiform layer, while the cells were still highly activated in the more severely affected inner retina (Fig. 3b, c).

Since expression data are normalized to housekeeper expression, higher RNA detection rates represent enhanced levels of respective gene expression and are not due to increased microglia numbers that occur concomitantly. In line with that, a stronger labeling intensity per microglial cell can be appreciated in the anti-AIF1 immunolabeling (Fig. 3a). Interestingly, significantly lower expression levels of Aif1 and Itgam in retinal microglia were observed in cells from XBD173-treated postischemic eyes at 3 dps—the time point of peaking microglia activation (Fig. 3d). Next, we investigated the effect of XBD173 treatment on the microglial differentiation into the M1 (pro-inflammatory) or M2 (regenerative) phenotype. Regarding M1 markers, we found a significantly less pronounced rise of F4/80 expression in microglia of XBD173-treated mice at 3 dps, while no change in Tnf transcript levels was observed (Fig. 3e). Moreover, we found that Arg1 was upregulated from 7 dps on in microglia irrespective of XBD173 treatment (Fig. 3f).

In summary, we found a dampened but robust microglial response to acute ischemia-induced retinal degeneration also in animals subjected to XBD173 treatment.

### Altered Müller cell gliosis after XBD173 treatment

**Fig. 4:**
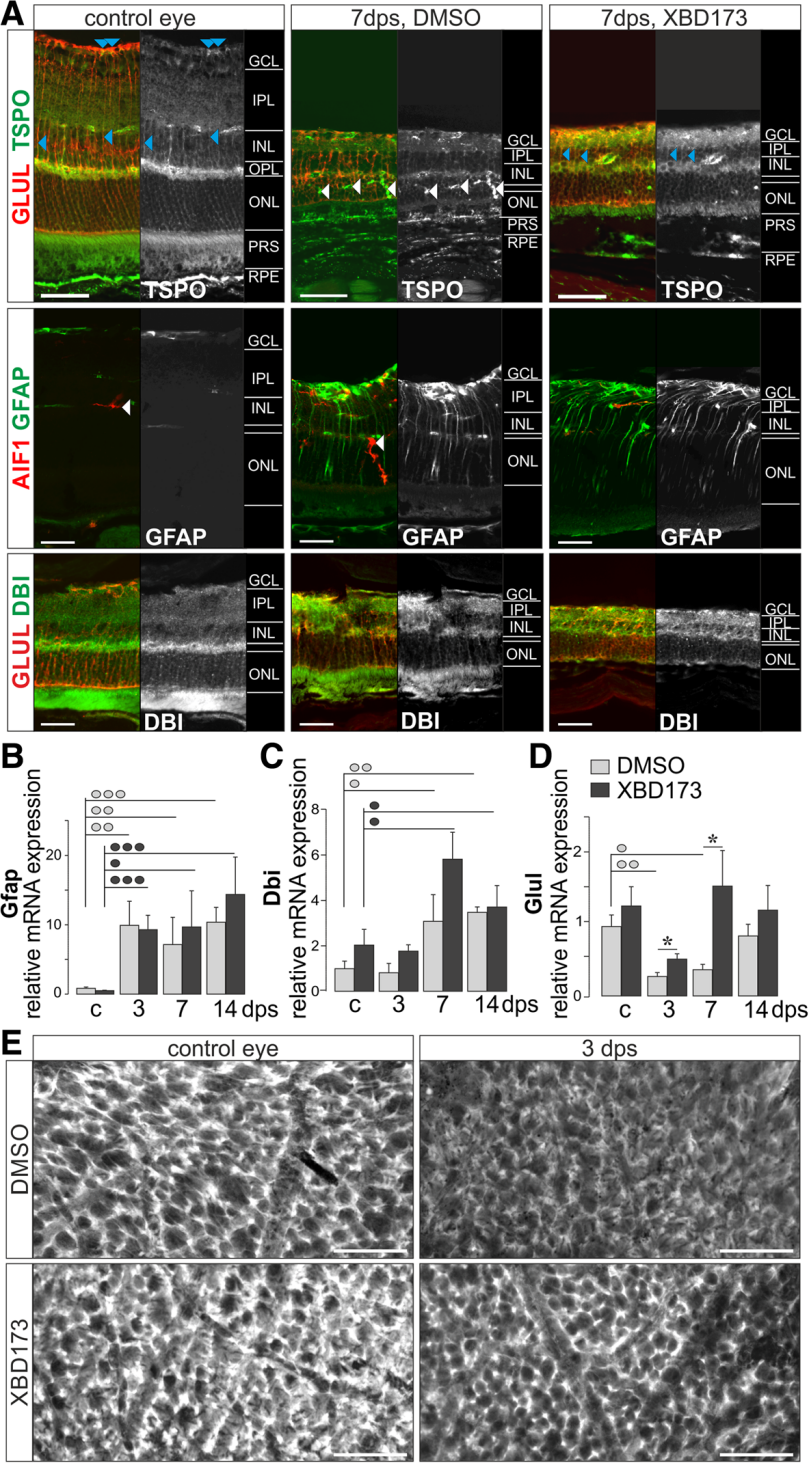
Müller glial reactivity in the postischemic retina. **a** Top, retinal slices from control and 7 days post-surgery (dps) eyes were labeled for TSPO and counterstained for the Müller cell marker glutamine synthetase (GLUL). Colabeling of TSPO and GLUL in Müller cell processes and end feet are pointed out by blue arrowheads. Middle, immunolabeling for the microglia marker AIF1 and glial fibrillary acidic protein (GFAP), a marker for Müller cell gliosis. Bottom, immunoreactivity for DBI partially colocalizes to that of GLUL. White arrowheads point at putative microglia. Expression of Gfap (**b**), Dbi (**c**), and Glul (**d**) was tested by qPCR from MACS-enriched Müller cells of control (c), 3, 7, and 14 dps. **b**–**d** Bars represent mean values ± SEM (*n* = 3–6 mice of each treatment group and time point). */°/•*P* < 0.05; °°*P* < 0.01; °°°/•••*P* < 0.001. The color of the circle indicates the treatment group. **e** Retinal flatmounts labeled for GLUL and focused on the ganglion cell layer (GCL) demonstrating reduced expression of GLUL at 3 dps which are more pronounced in postischemic retina of vehicle controls. **a**, **e** Scale bars, 50 μm

**Fig. 5:**
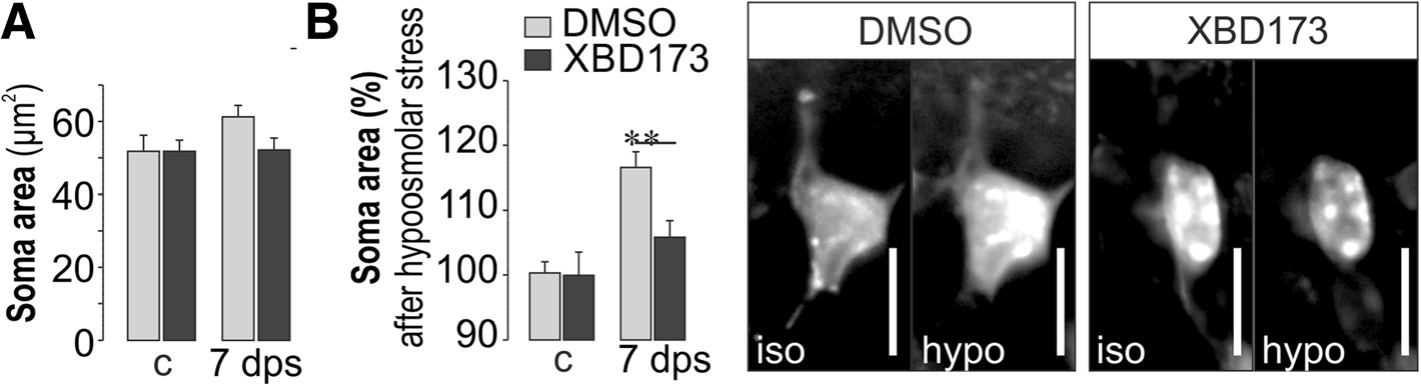
Müller cells maintain part of their homeostatic functions in the XBD173-treated postischemic retina. **a** At 7 dps, Müller cells from wild-type mice display a slight increase of the mean soma area determined in vital retinal slices on basis of labeling with Mitotracker Orange, which was not observed in XBD173-treated mice. **b** Left, the ability of Müller cells to maintain their volume constant was tested by challenging them with a hypoosmotic solution for 4 min. An efficient Müller cell volume regulation was significantly better preserved in XBD173-treated postischemic retinae 7 dps, while Müller cells from control eyes did not swell at all. Right, representative images from Müller cell somata loaded with Mitotracker Orange before and after 4 min of hypoosmotic stress. **a**, **b** Bars represent mean values ± SEM from 8 to 20 cells out of 2–3 animals. **P < 0.01. Scale bars, 10 μm

Given that Müller cells express the highest protein levels of TSPO in the healthy retina (apart from the also strong expression in the underlying RPE), we tested for specific effects of XBD173 treatment on them. Labeling for TSPO revealed no major alteration of its expression level in Müller glia 7 dps compared to the control eyes at protein level even though slightly higher mRNA expression had been detected (Figs. 2b and 4a). Immunoreactivity for the endogenous TSPO ligand DBI colocalized with the Müller cell marker glutamine synthetase but produced a rather diffuse labeling of spot-like structures across all retinal layers (Fig. 4a). No major changes in the DBI labeling intensity were observed in the postischemic retinae of both treatment groups 7 dps. Glial fibrillary acidic protein (GFAP)—a common marker of Müller cell gliosis—was upregulated in Müller cells of XBD173-treated retinae to a similar degree as in DMSO controls as early as 3 dps and stayed at a constantly high level until 14 dps as determined by immunolabeling (Fig. 4a) and quantitative PCR (Fig. 4b). Even though no obvious difference had been detected via immunolabeling, we also found a significant rise in Müller cell DBI expression at transcript level around 7 dps (Fig. 4c). Of note, DBI expression was not influenced by XBD173 treatment (Fig. 4c). Finally, we observed that glutamine synthetase, a key enzyme in the glutamate-glutamine cycle, was downregulated at 3 and 7 dps in Müller cells of vehicle controls in line with the findings from earlier studies [32] (Fig. 4d). At 14 dps, expression levels partially recovered. Interestingly, the reduction of glutamine synthetase expression in XBD173-treated mice was less pronounced at 3 dps and recovered significantly faster compared to vehicle controls. These findings are supported by a more intense glutamine synthetase labeling especially of the inner stem processes of Müller glia in postischemic retinae of animals from the XBD173 group at 3 and 7 dps (7 dps, Fig. 4a; for a better comparison, see scans focused to the plane of Müller cell end feet in retinal flatmounts at 3 dps, the time point of the most pronounced difference of Glul expression as indicated by qRT-PCR, Fig. 4e). Accordingly, we speculated that upon XBD173 treatment, some Müller glia functions were better maintained after transient ischemia. To follow up on this, we checked for a well-characterized feature of Müller glia typically affected by their gliotic response—their capability to counteract volume changes when challenged with hyposmotic stress for several minutes [33]. We found that the mean size of Müller cell somata of XBD173-treated mice at 7 dps was similar to that of the control eyes, while there was a trend to larger Müller cell somata in postischemic retinae of vehicle controls indicative of an altered gliotic response at 7 dps (Fig. 5a). Challenging retinal slices with a solution of 60% physiological osmolality, gliotic Müller cells from vehicle control postischemic retinae were not able to keep their volume constant. In contrast, the volume of those from the contralateral healthy eyes did not change (Fig. 5b). No significant Müller cell swelling was observed in the postischemic retina from XBD173-treated mice (Fig. 5b), suggesting that neuron-supportive functions of Müller cells are better preserved upon XBD173 treatment.

**Fig. 6:**
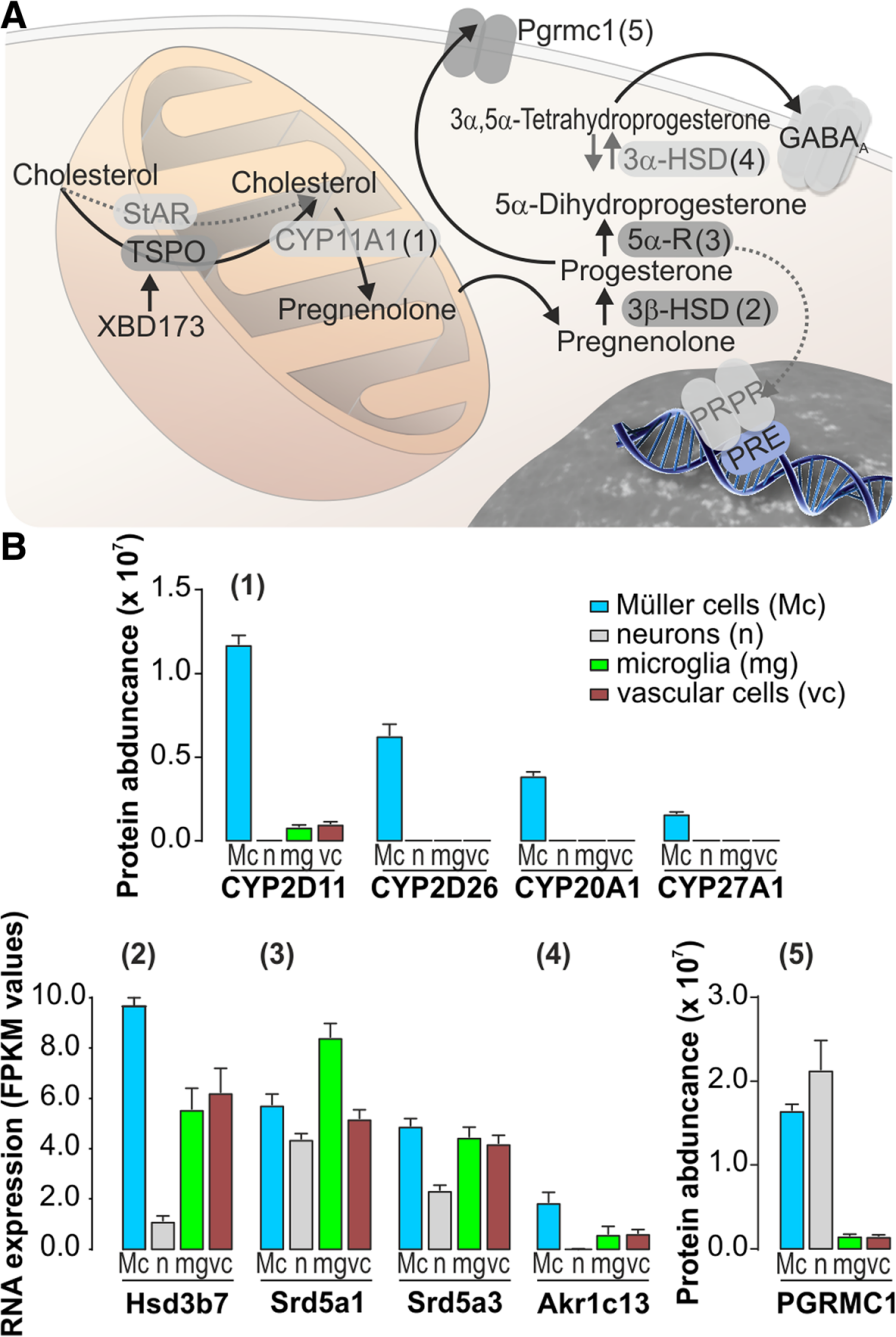
Expression of genes involved in neurosteroid biosynthesis in different retinal cell populations. **a** Scheme of neurosteroid synthesis from cholesterol where one rate-limiting step is its import into mitochondria. **b** Bar diagrams in (1) reflect the expression of the only cytochrome P450 family genes (Cyp2d11, Cy2d26, Cyp20a1, Cyp27a1) found to be expressed at protein level determined by quantitative LC-MS/MS mass spectrometry in the distinct retinal cell types. (2–4) Expression of enzymes for further conversion of pregnenolone to progesterone and other neuroactive steroids was low and only detectable at transcript level by RNA sequencing. (2) The highest expression of 3β-hydroxysteroid dehydrogenase (3β-HSD) type VII (Hsd3b7) were detected in Müller cells. (3) Two subtypes (Srd5a1, Srd5a3) of steroid 5α-reductase (5α-R) are ubiquitously expressed in all retinal cell types. (4) Akr1c13 belonging to aldo-keto reductase family 1 gene family (like 3α-hydroxysteroid dehydrogenase (3α-HSD)) is expressed at low levels and rather specifically in Müller glia. Fpkm, fragments per kilobase million. (5) High protein levels of the progesterone receptor membrane component 1 (PGRMC1) were detected in Müller glia and retinal neurons. Mc, Müller cells; n, neurons; mg, microglia; vc, vascular cells. Hsd3b7, 3β- and steroid δ-isomerase 7; Srd5a1, steroid 5 alpha-reductase 1; Srd5a3, steroid 5 alpha-reductase 3; Akr1c13, aldo-keto reductase family 1, member C13

Recently, we found that progesterone improved the efficiency of Müller cell volume regulation at nanomolar concentrations [34]. Considering the possible role of TSPO in neurosteroid (e.g., progesterone) synthesis [7, 11], it is tempting to speculate that XBD173 exerts part of its beneficial effects on Müller cells by enhancing retinal neurosteroid production. We checked whether retinal cell types express relevant genes and enzymes (Fig. 6). Steroidogenic acute regulatory protein (StAR), an alternative shuttle for cholesterol to the inner mitochondrial membrane [35], was not expressed at all (no transcript or protein was identified via RNA-seq or mass spectromic analysis, respectively). Thus, TSPO seems to be the only option to move cholesterol into the mitochondria in retinal cells. Similarly, no expression of Cyp11a1, typically metabolizing cholesterol to pregnenolone in mitochondria [36], was found on RNA or protein level in any of the retinal cell types. However, high levels of closely related cytochrome p450 family members such as Cyp20a1, Cyp27a1, Cyp2d11, and Cyp2d26 were expressed at high levels in Müller glia compared to other retinal cells (Fig. 6). Similarly, for 3β-hydroxysteroid dehydrogenase (3β-HSD; gene ID: Hsd3b1), needed for the generation of progesterone from pregnenolone, no reads for its transcript or unique peptides indicative for its expression were identified, while the closely related Hsd3b7 gene was strongly expressed in Müller cells (Fig. 6b). Two subtypes of steroid 5α-reductase (5α-R) are ubiquitously expressed (Srd5a1, Srd5a3; Fig. 6b), while 3α-hydroxysteroid dehydrogenase (3α-HSD, gene ID: Akr1c4) was not found to be expressed in any retinal cell type. However, we detected Akr1c13 belonging to the same gene family as being expressed in Müller glia (Fig. 6b). These enzymes are involved in further processing of progesterone to 5α-dihydroprogesterone and 3α,5α-tetrahydroprogesterone with various effects in the CNS [36]. Surprisingly, the classical progesterone receptor was not expressed at all, while high protein levels of the progesterone receptor membrane component 1 (Pgrmc1) were detected in Müller glia and retinal neurons (Fig. 6b). This may imply that progesterone effects in the retina are primarily mediated via Pgrmc1 and not the classical progesterone receptor.

### XBD173 reduces neurodegeneration in the postischemic retina

**Fig. 7:**
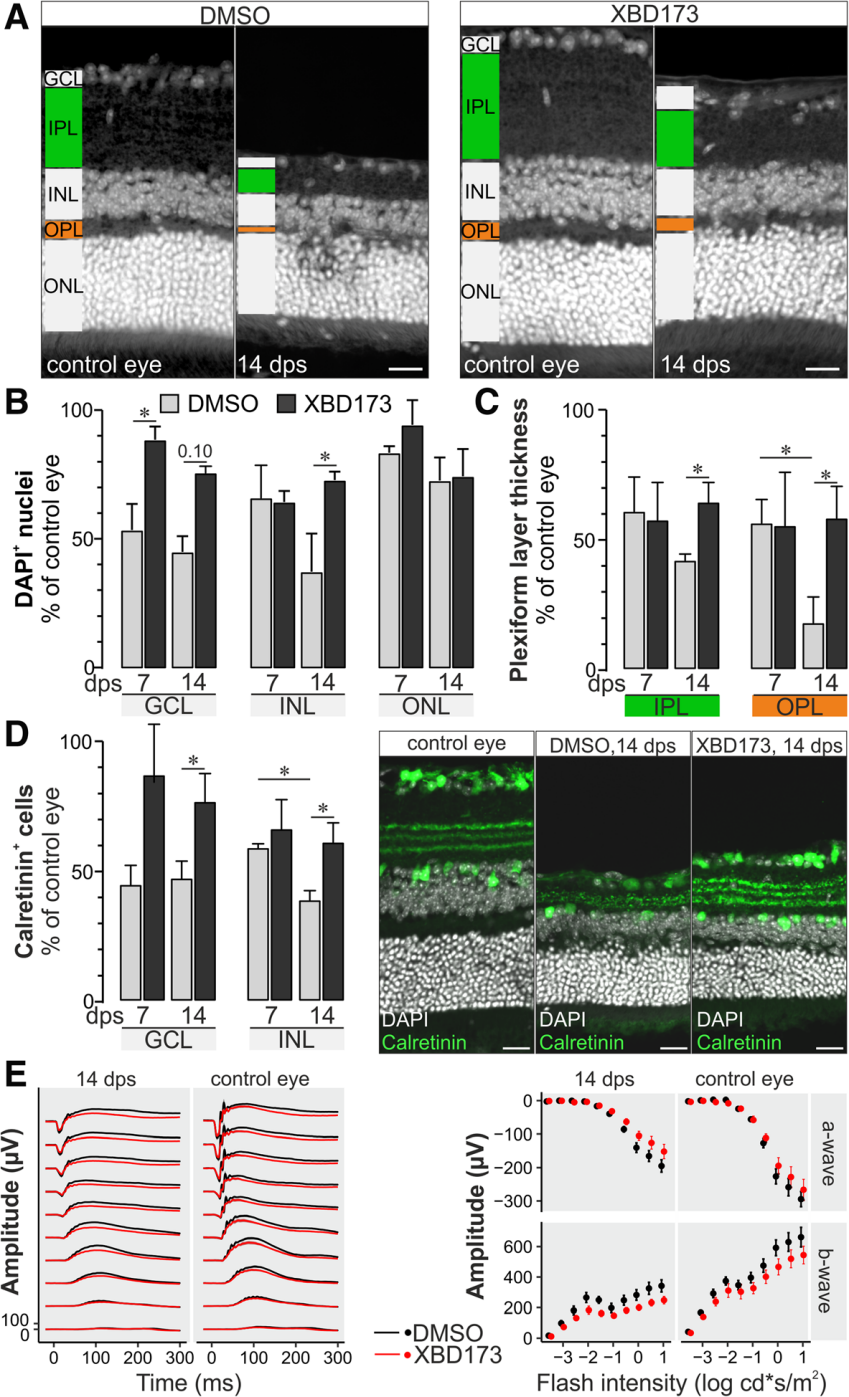
Characterization of the retinal phenotype after transient ischemia with or without XBD173 treatment. **a** Central retinal slices were stained with DAPI to visualize the nuclei. **b** Loss of retinal cells (*n* = 4–8 animals per group) in the different layers was quantified based on DAPI staining 7 and 14 days post-surgery (dps). **c** The thickness of the inner and outer plexiform layer (IPL and OPL) was measured in four central retinal slices from each retina. Bars represent values ± SEM from *n* = 3–4 of each treatment group. **d** Calretinin stainings (right) of the central retinal slices were used to quantify the survival rates (left) of ganglion and displaced amacrine cells (ganglion cell layer, GCL) and amacrine cells in the inner nuclear layer (INL). Bars represent values ± SEM from *n* = 3–4 of each treatment group. **e** Full-field electroretinogram recordings from mice 14 dps show a reduced responsiveness of retinae from XBD173-treated mice already in the control eyes. The relative reduction in light responsiveness after transient ischemia was similar in both treatment groups (*n* = 8 per group). Whiskers indicate the standard deviation. Scale bars, 20 μm

Finally, we characterized the XBD173 effect on neuronal cell death in our model of transient retinal ischemia which goes along with considerably thinning of the neurosensory retina (Fig. 7a). We found a significant protection especially of inner retinal cell types most severely affected by ischemic damage [6]. Fast degeneration was seen in cells of the ganglion cell layer which were reduced to 53.4 ± 10.2% at 7 dps in the vehicle controls but better survived in mice treated with XBD173 (88.5 ± 5.0%; Fig. 7b). This protective effect was still present at 14 dps. In contrast, little difference in cell loss was detected in the inner nuclear layer (INL) at 7 dps, while at 14 dps, a better survival of cells could be shown also in this layer (Fig. 7b). Given the consistently higher cell counts in the inner retinal layers of XBD173-treated mice, we asked whether this is also reflected by a better maintenance of synaptic structures. Analysis of the thickness of the inner and outer plexiform layers revealed that XBD173 treatment was protective for neurites. The thickness of both layers was significantly better maintained at 14 dps (Fig. 7c). To quantify ganglion and amacrine cells in the inner retina, we performed calretinin stainings. Analysis of this subset of cells largely mirrored the findings of the DAPI-based cell counts confirming the protective effect of XBD173 (Fig. 7d). Note that XBD173 treatment also preserved the intricate layering of calretinin-positive dendrites in the IPL. Photoreceptor degeneration occurred to a similar and comparably low level independent from the treatment regimen. Survival rates at 14 dps were still as high as 72.7 ± 9.0% in the vehicle controls and 74.3 ± 10.3% in XBD173-treated mice (Fig. 7b).

Lastly, we tested in electroretinogram (ERG) recordings whether the higher number of surviving neurons in treated mice resulted in a better retinal responsiveness to light stimuli. A- and b-wave amplitudes of control eyes of XBD173-treated mice were reduced to about 89.9% and 81.3%, respectively, compared to respective amplitudes measured in untreated animals (Fig. 7e). No differences between the level of ischemia-induced changes of retinal light responsiveness could be observed between the experimental groups (Fig. 7e).

## Discussion

For the first time and partially in contrast to previous studies [21, 22], we showed that TSPO protein expression was the highest in Müller and vascular cells, but low in microglia and almost absent in retinal neurons of the healthy retina (Fig. 2b). Additionally, we found a strong TSPO expression in RPE (Figs. 1c and 4a). TSPO in RPE cells was implicated to mediate cholesterol clearance from the subretinal compartment, and its age-related downregulation may promote progression of age-related macular degeneration [37]. In view of its high expression, TSPO function in RPE cells deserves further studies to elucidate its relevance in retinal disease.

Next, we investigated TSPO expression in postischemic retinae. Like others [22], we found a temporally defined upregulation of TSPO in microglia (and possibly macrophages) at 3 dps that declined at 7 and 14 dps (Fig. 3). With some delay, Müller cells significantly enhanced TSPO expression at 7 dps (Fig. 3b), corroborating the findings of TSPO upregulation in reactive brain astrocytes [9]. In contrast, no major change in TSPO expression was found in vascular cells at any time point post-surgery even though they express TSPO at high levels apparently relying on its function. Possibly, modulation of TSPO is associated with an active cellular response, as it occurs in microglia and Müller cells—those retinal cell types primarily shaping the retinal immune response. Considering that TSPO, among other mechanisms, influences the glial energy metabolism [16, 38], its upregulation in reactive glia may reflect an adaptation to their higher need for energy to rebalance the disturbed retinal homeostasis and to mediate an appropriate immune response. The energy metabolism of proinflammatory M1 microglia switches from mitochondrial respiration to primary glycolytic activity and in consequence fosters the generation of detrimental reactive oxygen species [39, 40]. It could be hypothesized that enhanced TSPO activity stabilizes the oxidative microglial metabolisms thereby promoting their polarization towards the protective M2 phenotype. Note that Müller glia normally generate ATP via anaerobic metabolic pathways [41], which possibly does not suffice under pathological conditions so similar beneficial bioenergetic effects of TSPO stimulation may account for the improved neurosupportive phenotype of Müller glial upon XBD173 treatment in our retinal ischemia model.

Accordingly, we further characterized in-depth the glial response pattern. Less microglia accumulated in XBD173-treated postischemic retinae and the microglial activation profile implied a trend towards an M2 regenerative phenotype (Fig. 3e, f) confirming recent studies demonstrating that XBD173 reduces the microglial neurotoxicity, migration, and proliferation [21, 22]. It should be emphasized that even if a treatment regimen was applied as used by others [24], XBD173 effects on microglia were only moderate and hallmarks of microglial activation (rising cell numbers, upregulation of TSPO and pro-inflammatory markers, morphological alterations), even though partially dampened, were still observed in treated animals (Figs. 2 and 3). Possibly, this is because transient ischemia disturbs retinal neurons and glia leading to more severe imbalances of the retinal homeostasis than in disease models investigated previously. Consequently, the retinal immune response including that of microglia needs to be more powerful than upon selective photoreceptor loss after light damage [24] or LPS-induced inflammation [22] and thus partially outruns XBD173 effects.

TSPO in Müller cells has not been investigated to date. Given that they express highest levels of TSPO protein compared to all interrogated cell types in the healthy retina, it was not surprising to find significant effects of XBD173 on these major retinal macroglia. XBD173 treatment led to a trend of higher expression levels of the endogenous ligand DBI in Müller cells (Fig. 4c) so that beneficial effects of enhanced TSPO function possibly were reinforced. In addition, DBI was upregulated in gliotic Müller cells after transient ischemia confirming findings from a model of LPS-induced retinal degeneration [22]. Like in microglia, XBD173 did not completely abolish Müller cell gliosis as a robust upregulation of GFAP, and a temporary downregulation of glutamine synthetase was found (Fig. 4). However, further analysis revealed that key features essential to maintain the neuron-supportive microenvironment were significantly better preserved in Müller cells upon XBD173 treatment. Reduction of glutamine synthetase expression was less pronounced and recovered faster (Fig. 4d, e). This Müller cell-specific enzyme is key for the glutamate-glutamine cycle and, hence, for neurotransmitter recycling [42]. Its activity fosters glutamate uptake [43] thereby preventing neurotoxic effects of glutamate [32, 44–46], and recently, its relevance to maintain the blood-retinal barrier has been discussed [47]. Additionally, we found that the highly efficient cell volume regulation of normal Müller cells stays active in Müller cells of postischemic XBD173-treated retinae but is lost in gliotic Müller cells of vehicle controls (Fig. 5). This Müller cell feature is considered as a prerequisite for their role in stabilizing the retinal ion and volume homeostasis [1–3] and, thus, neuronal function. The role of TSPO in the neurosteroidogenesis was intensively studied [7, 11, 13]. Given (i) the high expression levels of TSPO and downstream enzymes for neurosteroid synthesis in Müller glia (Figs. 1 and 6) together with (ii) beneficial effects of neurosteroids on Müller cell volume regulation [34] as well as recent reports (iii) demonstrating beneficial effects including a reduced gliotic activation of Müller glia upon treatment with the progesterone analogue Norgestrel [48], the effect of XBD173 on Müller cell volume regulation and gliotic activation pattern might be partially due to the enhanced neurosteroid synthesis. An augmented retinal allopregnanolone generation upon TSPO stimulation in retinae was proven recently [23].

Having identified an altered glial response pattern upon XBD173 treatment that possibly fosters better neuronal survival, we revisited the question of whether the TSPO ligand mediates neuroprotection in the postischemic retina. Indeed, we found an improved neuronal survival in XBD173-treated postischemic retinae. Pronounced effects were observed for inner retinal neurons and neuronal dendrites (Fig. 7) consistent with our earlier studies describing protective effects of a better maintained Müller cell homeostatic function on the survival of these specific cell types [6]. This strengthens our hypothesis that cells generating action potentials especially rely on (i) a tight glial control of glutamate levels and a (ii) well-balanced ion and volume homeostasis. Of note, we found strong TSPO expression also in the RPE (Fig. 1). However, since dominant XBD173 effects were observed for neurons of the inner retina (Fig. 7), a contribution of the RPE is rather unlikely but cannot be completely excluded.

The functional readout via ERG recordings was difficult to interpret regarding the changes in the postischemic retina as XBD173 dampened a- and b-wave amplitudes even in healthy control eyes (Fig. 7). A likely explanation is an enhanced generation of neurosteroids upon XBD173 treatment (possibly in Müller glia) acting on GABA_A_ receptors thereby reducing the retinal light response acting on inhibitory retinal synaptic pathways [49–51]. Similarly, reduced retinal responses were described if individuals were treated with the GABA_A_ agonist diazepam [52]. In fact, enhanced GABA_A_ receptor activation, e.g., on dendrites of bipolar cells [52, 53], may contribute to the neuroprotection mediated by XBD173. However, since the cellular composition of the postischemic retina changed together with the XBD173 effect on retinal signaling that possibly differently impacts on specific retinal subtypes, no conclusion could be drawn from ERG of the postischemic eyes. Future experiments should elucidate whether a washout of XBD173 over several days leads to restoration of normal responses in the healthy control eye and possibly also detectable differences between postischemic eyes from the DMSO and XBD173 group.

## Conclusion

The agonistic TSPO ligand XBD173 significantly reduces the neuronal cell loss in retinae following transient ischemia. Given the predominant expression of TSPO in micro- and Müller glia in the retina and its absences from retinal neurons, XBD173 primarily affects glial cells. The distinct contribution of these two glial entities to XBD173-mediated neuroprotection needs further investigation. Our results contribute to the increasing body of evidence that points at a putative therapeutic use of TSPO agonists for the treatment of retinal.

## Abbreviations

3α-HSD: 3α-Hydroxysteroid dehydrogenase
3β-HSD: 3β-Hydroxysteroid dehydrogenase
5α-R: Steroid 5α-reductase
Aif1: Allograft inflammatory factor 1
Arg1: Arginase 1
CNS: Central nervous system
DBI: Diazepam-binding inhibitor
DMSO: Dimethyl sulfoxide
ERG: Electroretinogram
F4/80: Adhesion G protein-coupled receptor E1
GABA: Gamma-aminobutyric acid
GCL: Ganglion cell layer
GFAP: Glial fibrillary acidic protein
Glul: Glutamine synthetase
INL: Inner nuclear layer
IPL: Inner plexiform layer
Itgam: Integrin subunit alpha M
ONL: Outer nuclear layer
OPL: Outer plexiform layer
qRT-PCR: Quantitative real-time polymerase chain reaction
RNAseq: RNA sequencing
RPE: Retinal pigment epithelium
StAR: Steroidogenic acute regulatory protein
TNFα: Tumor necrosis factor alpha
TSPO: Translocator protein 18 kDa
XBD173: Emapunil, *N*-Ethyl-7,8-dihydro-7-methyl-8-oxo-2-phenyl-*N*-(phenylmethyl)-9H-purine-9-acetamide
